# Supplementary material for: Perioperative changes of response to antiplatelet medication in vascular surgery patients
Source: PLoS One. 2020 Dec 29;15(12):e0244330. doi: 10.1371/journal.pone.0244330 (PMC7771706; doi:10.1371/journal.pone.0244330)
Supplement: Attachment — Submitted filename: 2020_11_13 Response to Reviewers.docx [file pone.0244330.s002.docx]

Response to the academic editor´s and reviewers comments

PONE-D-20-25881

Perioperative changes of response to antiplatelet medication in vascular surgery patients

Dear Prof. Raffaele Serra,

Thank you very much for the detailed reviewer’s critique of our manuscript new entitled „Perioperative changes of response to antiplatelet medication in vascular surgery patients”. We have reformatted our manuscript according to the PLOS ONE's style and reference style requirements. Furthermore we provide additional information about the participant recruitment and a sample size and power calculation in the methods section. Moreover, added a post hoc adjustment for multiple comparisons (Holm-Bonferroni method) and discuss major limitations of this study in the end of the manuscript. Please find attached our revised manuscript and our counterstatement to the reviewers’ comments.

We hope to have addressed the reviewers’ comments in an adequate manner and deeply hope that this substantially revised manuscript will be considered for publication in PLOS ONE.

Sincerely, yours

Thomas Hummel

**Response to the comments of the academic editor:**

1. Please ensure that your manuscript meets PLOS ONE's style requirements, including those for file naming.

PLOS ONE´s style requirements are added and carefully implemented in the manuscript.

2. In your Methods section, please provide additional information about the participant recruitment method and the demographic details of your participants. Please ensure you have provided sufficient details to replicate the analyses such as: a) the recruitment date range (month and year), b) a description of how participants were recruited, and c) descriptions of where participants were recruited and where the research took place.

The recruitment of the participants at the time of inpatient admission took place at the department of vascular surgery of the St. Josef Hospital, university hospital of the Ruhr-University of Bochum from February 2010 to December 2011. The study was designed as a prospective observational study and part of a medical doctoral thesis of the Ruhr-University of Bochum. The time of the patient´s selection was associated to the graduator and his temporary presence in the clinic. Therefore the recruitment time period was prolonged and the time for statistical analysis was also extended. Nevertheless we believe that our data are consistent and important to understand periprocedural changes in response of antiplatelet medication in vascular surgery patients.

We added recruitment information, demographic detail and date range of the study to the Methods section (page: 3+4, lines 91-110)

3. Please provide a sample size and power calculation in the Methods, or discuss the reasons for not performing one before study initiation.

The sample size planning was performed using a significance level of α=0,05, estimated Pearson´s correlation coefficient of r=0,25 and stochastic power of 0,8. The sample size of the study calculated in this way was n=123 in order to get a significant result. The sample size and power calculation is added in the Methods. (page: 6, lines 165-168)

4. Please include additional information regarding the survey or questionnaire used in the study and ensure that you have provided sufficient details that others could replicate the analyses. For instance, if you developed a questionnaire as part of this study and it is not under a copyright more restrictive than CC-BY, please include a copy, in both the original language and English, as Supporting Information.

There was no questionnaire used in the study. All additional information regarding the survey is accessible in the publication.

5. Please ensure you have thoroughly discussed any potential limitations of this study within the Discussion section, including the potential impact of confounding factors.

We added limitations of our study in the Discussion section (page: 14+15, line: 375-405)

6. PLOS requires an ORCID iD for the corresponding author in Editorial Manager on papers submitted after December 6th, 2016.

The ORCID iD for the corresponding author (ORCID iD: https://orcid.org/0000-0001-6931-5930) is added in the Editorial Manager.

**Response to the comments of reviewer #1:**

1- Abstract, page 2, lines 34-35: The sentence describing high or low response for platelet activity is awkward and difficult to understand- please revise/clarify further.

This sentence is clarified and the abbreviations are explained later on in the manuscript. (page: 2, line: 34-35)

2- Abstract, page 2, lines 52-54: There is a comment about ALR increasing, did CLR change?

Results of CLR were added. (page: 2, line: 54-55)

3- Abstract, page 2, line 51- diabetes and insulin are confounded by indication—they must be somewhat co-linear and should likely not be reported separately as independent risk factors.

These are related factors, so they are connected in the abstract, also post hoc adjustment changes significance level and results (page: 2, line52-53).

4- Introduction, page 3, lines 65-78: It would be helpful if the authors could cite peer reviewed evidence about treatment failures that are ascribed to failure in antiplatelet therapy- can they provide prevalence information that is linked to actual clinical events/outcomes? This would further highlight clinical (and not experimental) significance and importance of studying platelet activity in vascular surgery patients.

The Introduction section has been revised and the useful, but small, amount of evidence regarding surveillance and outcome was added. (page: 3, line: 71-76)

5- Methods, page 4, lines 97-102: for the described exclusion criteria- please define renal impairment. Clarify ‘recent’ GI bleeding history. What is ‘severe’ cardiac disease?

Reasonable and understandable objection to the described study exclusion criteria. Thank you for that. This prospective study was designed as a observational study, so that the exclusion criteria were limited to general contraindications to participation in a scientific study (as cognitive disorders, pregnancy…), and contraindications to the study medication or intrinsic activation / or depression of platelet activity (Gastrointestinal ulceration with history of bleeding within six weeks, NYHA stage 4, kidney or liver failure, abnormal platelet count, known coagulation disorders). The exclusion criteria have been newly explained and specified in the manuscript. (page : 5, line: 127-133)

6- Methods- Inclusion/Exclusion criteria- please present a flow diagram demonstrating the total denominator and ultimate numerator of patients included/excluded.

To our regret, we cannot present a flowchart for patient inclusion and exclusion. The study was designed as an observational study. Moreover, patients were recruited in the daily clinical routine. Therefore we have not detected all other patients of our department. (See response to Point 5 and 7)

7- Methods- how were patients selected- was it only those seen in the clinic? Was there a group who undergo surgery vs group not undergoing surgery - may help for trying to establish baseline comparisons [controls]- please clarify.

The recruitment of the patients was at the time of inpatient admission and was designed as a observational study and part of a graduation as a medical doctor of the Ruhr-University of Bochum. Therefore the recruitment was discontinuous, as it is unfortunately common in this setting. There was no planed control group in this study. Each participant had a symptomatic vascular disease with an indication for vascular treatment with different degrees of invasiveness (endovascular / local TEA / peripheral bypass). We hypothesized that low response is increased after vascular therapy in common vascular procedures, with regard to the invasiveness of the procedure. The recruitment information was added in the methods section. (page: 3+4; line: 91-95)

8- Results, page 14- title of Figure 2 has a typographical error.

The typographical error has been corrected. (Fig 2). We apologize for this.

9- Results- page 15-16: The authors need to describe the types of operations that the patients underwent and differentiate platelet activity based upon the types of procedures.

The changes of response to ASA in the different groups of operations are added (page: 9+10, line: 248-258)

Procedures are now described in the methods section (page: 4, line 103-109)

10- Results- is there any clinical outcome information- adverse thromboembolic complications that occurred in the patients?

We totally agree with reviewer #1. Clinical outcome information or the detection of thromboembolic complications would be very interesting to evaluate the impact of ALR /CLR in patients outcome after different vascular procedures. Unfortunately, no outcome or follow up data were collected from the patients. The aim of our study was the detection of changes in antiplatelet response in perioperative comparison. We believe this study could be the basis for further investigations with clinical endpoints and follow up platelet response measurements. Moreover we discuss this interesting issue in the limiations section. (page15, line 393-405)

11- Discussion- can the authors provide a deeper discussion surrounding how if validated, this information will be used for point of care testing and clinical decision making?

The impact of our periprocedural findings in clinical context and our daily practice of testing is explained at the end of the Discussion section. (page: 15, line: 405-409)

**Response to the comments of Prof. Dr. Steven M. Frank / reviewer #2:**

The authors should put forth a hypothesis being tested, otherwise it looks more like the authors are just looking for statistically significant relationships among variables.

We formulated our hypothesis at the end of the introduction section. (page: 3, line: 84-86)

The abstract, and maybe even the title should specify the study design as prospective observational (I think that it correct).

We specified the study design in the introduction section of the abstract. (page: 2, line: 38)

The abstract is not written clearly. The first sentence is the most problematic with the “/” and the 4 abbreviations all in one sentence. The authors should simply tell us what the issues are and why this study is being done before throwing a confusing sentence at the reader. A hypothesis should be included in the abstract - at the end of the introduction. Page 2 – LM 43 – risk factors for what? Page 2 – LM 48 – add “ taking antiplatelet medications aspirin and/or clopidogrel”. Almost all good papers have at least one P value in the abstract and this paper does not.

The first sentence of the abstract is clarified and the abbreviations are explained later on in the manuscript. (page: 2, line: 34-35). We formulated the hypothesis at the end of the introduction of the abstract (page: 2, line: 38-39) and specified the risk factors (page: 2, line: 43-44). At page 2 line 49 we added “ taking antiplatelet medications aspirin and/or clopidogrel”. P value was added for changes in response. (page: 2, line: 54).

Page 3 – LM 73 – Several times the authors use the term “common vascular patients”. This should read “undergoing common vascular procedures”. A procedure can be common, but a patient cannot.

The term “common vascular patient” was changed in the whole manuscript and even in the title.

Page 3 – LM 87 – Here it is fine to say “common vascular procedure”, however in the methods section the authors need to tell the reader what these procedures are. Angioplasty, angiogram, stent placements, open surgery, endovascular surgery, etc.

The performed procedures are explained in the methods section. (page: 4, lane: 103-109)

Page 5 – LM 133 – Is it clearly evident to use these specific cutoffs to dichotomize patients into responders and non-responders?

The cut off values used for the detection of ALR and CLR are part of the Multiplate instruction for use. The cut-off value >46 U of the ADPtest for CLR is well established and underlaid with good evidence and clinical outcome measurement based on the outlined reference (Sibbing D. et al. n=1608 cardiology patients undergoing intervention with increased risk in comparison to normal responders). In the detection of ALR different cut-off values are recommended by the manufacturer and used in the literature (Roche Diagnostics Multiplate). For the detection of a strong inhibition of cyclooxygenase (COX) -1 by ASA is recommended <30 U as cut-off (based on a study with n=76 by Pape et al. 2007). For the detection of an adequate inhibition of COX-1 by ASA <40 U is recommended as a cut-off. These cut off is based on the results of the stated study in the manuscript by Al-Azzam et al. with n=418 participants. However, no information is given on possible areas of application of the different cut-off values for ALR and there are no clinical outcome studies available based on the different cut off values with the range from 30 to 40 U for ASA used in the literature. In the present study, the ASA response was classified using the cut-off <40 U. The lack of evidence and different cut-off values used in the literature in the detection of ALR using Multiplate represents a limitation of the study, which was added in the limitations section at the end of the discussion (page: 14, line: 375-381).

Are there interactions between the response to aspirin and clopidogrel that would confound the results of this study? Since 84% of patients were on both, can we truly be sure the aspirin effect is not confounded by the other drug?

There were 148 (84.1%) patients on medication with 100 mg aspirin, 12 (6.8%) on dual antiplatelet medication with 100 mg aspirin and 75 mg clopidogrel, 11 (6.3%) on monotherapy with 75 mg clopidogrel, two (1.1%) on dual antiplatelet medication with 300 mg aspirin and 75 mg clopidogrel and 3 (1.7%) with 300 mg aspirin included in this study. Because of the small number of patients on dual medication, we can rule out any relevant influence on the presented results

Table 1 – The rule of significant digits should be followed. Unless age was collected to the precision of 0.01 years, then the mean and SD do not get reported to this degree of precision. See the New England Journal for example, where almost all papers report age in whole numbers – no decimal places. Percentages are usually reported to the 0.1 degree of precision. P values are not given to 4 places after the decimal point in good journals. Usually 2 significant digits is the practice.

Tables were revised and in the text accordingly corrected.

Does it matter if patients were on enteric coated vs. regular aspirin? If so, this should be added.

There is little evidence, that enteric coated ASA and regular aspirin as clopidogrel hydrogen sulfate and clopidogrel besylate could have different impaired antiplatelet effect. In our study we had a homogenous medication with regular aspirin and clopidogrel hydrogen sulfate. Therefore we only used aspirin nomenclature in the text. The confusing enteric labeling of the tables has been changed. (see titles table 1 & 2).

One major concern is that there were 30 variables assessed for their relationship to platelet responsiveness, but no post hoc adjustment for multiple comparisons (e.g. Bonferroni). Taking this into account, a P value of 0.00167 would be required for significance, otherwise we will see false positive findings. 2 of 3 findings in table 2, and 3 of 4 findings in table 3 would thus change to being not significant.

We added the post hoc adjustment for multiple comparisons (Holm-Bonferroni method). Therefore initial findings in the risk factor analysis changes and were added in the results section. Thus the discussion was adapted to the findings in the post hoc analysis. (page : 7, line: 204-209) (page: 10, line: 252-254) (page: 10, line: 255-2262)

Page 8 – LM 192 – This finding really should have a P value as it seems like one of the main findings in the paper.

Good and reasonable point. Thanks for that. P value was added to this main finding. (page: 9, line: 224-225)

Page 8 – Several times we see “p<0.05” but the text should give the actual p value, especially given the issue with multiple comparisons mentioned above.

The actual p values with addition of post hoc adjustment for multiple comparisons have been added in the manuscript.

Page 11 – LM 253-255 – This sentence should be part of a limitations paragraph, which, by the way, is missing and should be added.

We added limitations of our study in the discussion section. (page: 14+15, line: 375-404)

Discussion – It would be most interesting to expand on proposed mechanisms for the decreased responsiveness to anti PLT medications after the procedures.

The discussion was changed, expanded and rearranged according to the post hoc analysis. (page: 13, line: 329-331) (page: 14, line347-361)

Verify Now is no longer from Accumetrics, it is now from Instrumentation Laboratories. Add Bedford, Massachusetts as well.

Correct. We are sorry. Actual information for VerifyNow is added. (page: 13, line: 322)

In the conclusion, it would be more impactful and clear to say the percentage of patients who were low responders approximately doubled postoperatively, or say there was an approximate 2-fold increase (13.13% to 27.5%).

We added the more impactful statement in the conclusion. (page: 16, line413-415)

I don’t see a figure legend. Is figure 1 showing preop or postop findings? A legend is needed.

Figure legends have been added.

Figure 2 – same thing. “pot-OP” should be “post-OP”. Numbers should have one digit after the decimal point.

Figure 2 has been corrected.
