# Supplementary material for: Perioperative changes of response to antiplatelet medication in vascular surgery patients
Source: PLoS One. 2020 Dec 29;15(12):e0244330. doi: 10.1371/journal.pone.0244330 (PMC7771706; doi:10.1371/journal.pone.0244330)
Supplement: Attachment — Submitted filename: 2020_12_06 Response to Reviewers.docx [file pone.0244330.s003.docx]

Response to the reviewers comments

PONE-D-20-25881

Perioperative changes of response to antiplatelet medication in vascular surgery patients

Dear Prof. Raffaele Serra,

Thank you very much for the reviewer’s comments of our manuscript entitled „Perioperative changes of response to antiplatelet medication in vascular surgery patients”. We feel grateful and believe that our manuscript has been significantly improved throughout the entire review process. Please find attached our revised manuscript and our counterstatement to the reviewers’ comments.

We hope to have addressed the reviewers’ comments in an adequate manner and deeply hope that this revised manuscript will be considered for publication in PLOS ONE.

Sincerely, yours

Thomas Hummel

**Response to the comments of reviewer #1:**

1- Abstract, Introduction: 'Reduced antiplatelet activity of aspirin' is abbreviated as 'ALR' however in the final sentence of the introduction to the abstract, the authors have 'ARL'.

We changed and clarified to the explained abbreviations in the final sentence of the introduction section of the abstract. (page: 2; line: 38)

2- Abstract, Results- 2nd sentence highlights 'The prevalence at the time of admission'-- what prevalence are they referring too exactly- is it any evidence of reduced antiplatelet activity or was there a specific threshold from the assay that determined a 'yes' or 'no' to the presence of reduced antiplatelet activity. Please clarify.

Important point. We clarified this sentence in a short way for the abstract (page: 2, line 48+49). Specific threshold and cut off values for the Multiplate aggregometry were explained in the materials and methods section in the manuscript (page: 6, line: 154-156).

3- Abstract, results line 52-53: the syntax/presentation of the sentence is awkward- please consider revising to clarify the results to 'The overall ALR increased significantly postoperatively to 27.5% (p=0.0006); however, there was no significant change in CLR that was detected.'

We changed and clarified this sentence. (page: 2; line: 51+52)

4- Discussion, line 273- the authors site a ALR rate of 13.1% but CLR rate of 20%; however, in the abstract, the CLR rate is 32% (line 50)- please clarify.

Astonishing that despite extensive intern text revision, such obvious errors are still overlooked. We are very sorry and glad that we have the opportunity to revise this mistake in the discussion section. Thank you for that. CLR rate was corrected to 32% and discussion was modulated. (page: 11; line: 268 + page: 21; line: 276-279))

5- Discussion- line 273-274- the comment that the patients were 'already without effective protection against thromboembolic events before the necessary vascular intervention'-- I think I understand what the authors are intimating but without linkage clinically (either through peer reviewed literature- which the authors highlight is largely missing in the literature, or by evidence in the current study- which we don't have enough clinical events )- can you actually make this statement? While hypothetically at higher risk, without actual peer reviewed evidence and/or clinical end-points in the current analysis, can this statement be scientifically supported?

Reasonable and understandable objection to the clinical impact of low response. We agree that the induced clinical outcome association of low response is inappropriate at this point of the text. The sentence was defused and corrected. (page: 11; line: 267-270). Later on in the discussion section, the evidence of the clinical impact of a low response is discussed. (page: 14, line 348-359)

6- Discussion lines 320-322: what is the difference between 'LR' and 'ALR'?

We clarified and changed LR (any low response to antiplatelet medication) to ALR (aspirin low response). (page: 13; line: 315-318)

7- Given the prevalence of aspirin/clopidogrel resistance- do the authors propose routine platelet aggregometry assays for all vascular patients undergoing intervention? It is unclear what without direct linkage to major adverse clinical events how to use the current findings in clinical practice. I think the observation is notable and I agree with the authors that additional study is warranted.

We totally agree to this objection. At the end of the discussion, we comment on this topic and also disclose our behavior in our daily practice. (page: 15+16, line: 389-404)

8- Can the authors provide an explanation as to why aspirin resistance increased postoperatively but Plavix did not?

Unfortunately, the informative value of the study with regard to clopidogrel is limited due to the small number of patients in this group. This point was taken into account in the evaluation of the results, discussion and the study limitations (page: 15, line: 385-388 + page: 12, line: 276-279).

**Response to the comments of Prof. Dr. Steven M. Frank / reviewer #2:**

Abstract L51-53: P-values should be included in the abstract when describing risk factors for ALR (insulin and CRP).

P-values were added to the results section of the abstract. (page: 2; line: 50+51)

Introduction L90-92: The phrase “with regard to the invasiveness of the procedure” is confusing in the hypothesis. Are the authors hypothesizing that more invasive procedures will lead to a higher incidence of low response rate? Please clarify.

We clarified the hypothesis at the end of the introduction section. (page: 3; line:80-82)

Methods L96-101: These are results (demographic and clinical characteristics) and should be moved to the Results section of the manuscript.

These patients characteristics were moved to the beginning of the results section. (page: 6; line: 170-173)

Discussion L443-451: This paragraph on the clinical significance of the results and determining who should receive these tests is, in my opinion, a very important point that needs to be expanded upon in the Discussion. In reading the manuscript, a big question that remains is, “what does this mean for patients?” While the 2-fold increase in low responders to aspirin is striking, it is important to discuss this in terms of what it means for patient care and clinical outcomes. Would you consider the patients you enrolled in this study “high risk,” and thus justified in receiving these tests? I encourage the authors to further address these points.

Important note, we expanded the end of the discussion section and clarified our point of view in terms of patient´s testing. (page: 15, line: 389-404)

Table 3: What does the row “Operative Procedure” under the Treatment subcategory refer to in this table? This is not part of the 5 groups that were discussed in the Methods section for operative approach. From the manuscript, it seems like this should be rephrased to “Open Operative Approach.”

We corrected table 3 and rephrased treatment subcategory. (table 3)
